# Fishers’ knowledge detects ecological decay in the Mediterranean Sea

**DOI:** 10.1007/s13280-020-01452-3

**Published:** 2021-01-16

**Authors:** Benedetta Veneroni, Paul G. Fernandes

**Affiliations:** School of Biological Sciences, Zoology Building, University of Aberdeen, Tillydrone Avenue, Aberdeen, AB24 2TZ UK

**Keywords:** Biodiversity, Fisheries, Local ecological knowledge, Mediterranean, Shifting baseline syndrome, Trawling

## Abstract

The Northern Adriatic Sea (NAS) is one of the most overexploited marine ecosystems in Europe. Given the gaps in scientific knowledge regarding the NAS, this study sought Fishers’ Ecological Knowledge (FEK) to determine NAS’ historical baselines for conservation. By interviewing 53 fishers in three ports of northern Italy, estimates of the catch rates of four commercial demersal species were generated over a 60-year period, and perceptions of target and non-target species’ diversity and benthic diversity were analysed in three groups of fishers (i.e. novices, experienced and veterans). Results showed a significant decline in perceived abundance of sole (*Solea solea),* common cuttlefish *(Sepia officinalis)* and mantis shrimp *(Squilla mantis)*, and evidence was found of a Shifting Baseline Syndrome (SBS) among novices. Given FEK’s ability to complement scientific knowledge, fishers’ participation in marine management policies and intergenerational communication should be enhanced, to improve the status of marine ecosystems and hinder SBS.

**Electronic supplementary material:**

The online version of this article (10.1007/s13280-020-01452-3) contains supplementary material, which is available to authorized users.

## Introduction

Located in the Mediterranean Sea, the Northern Adriatic Sea (NAS) is characterised by shallow depths (average of 33.5 m), sandy-muddy bottoms, strong vertical stratification and highly variable sea surface temperatures, with peaks of 5 °C in winter and 27 °C in summer (Russo and Artegiani 1996). The region has, by far, the highest annual production of demersal species (around 44,000 tons) in the Mediterranean (Mannini and Sabatella 2015; STECF 2016) and the highest number of trawlers (1043) of any European country (Colloca et al. 2017). The NAS presents the lowest probability of being sustainably fished (Colloca et al. 2017), and more than 90% of its marine resources are depleted (Lotze et al. 2011). Analytical stock assessments of some of the most common commercial stocks fished by Northern Adriatic bottom-trawlers have been conducted, but these estimate population dynamics only as far back as the mid 2000s (STECF 2019). In most cases estimated fishing mortalities (F) have been, and are, higher than the those considered to be sustainable (F_MSY_), such as in the case of red mullet, *Mullus barbatus* (F/F_MSY_ ratio of 1.2 in 2018); in others they are very much higher, as for sole, *Solea solea* (ratio of 3.0); and mantis shrimp, *Squilla mantis* (3.33).

The substantial fishing pressures incurred on the NAS were intensified by Italian fishers’ adoption of bottom trawl gears, notably beam trawls, otter trawls (also called *tartana*) and the *rapido* trawl, from the start of the 20th Century (Fortibuoni et al. 2017). Of these, the *rapido* is considered to exert the most disruptive effects (Pranovi et al. 2001), with its rigid mouth and iron teeth penetrating into the sediment (Giovanardi et al. 1998). The long-term use of the *rapido* and other trawls for demersal fishing is considered to have negatively affected not only the target species (Barausse et al. 2011; Damalas et al. 2015) but also non-target by-catch species such as elasmobranchs, marine mammals and diadromous fish (Abdul Malak et al. 2011; Giani et al. 2012). Furthermore, the *rapido* has exerted substantial pressure on the seabed and benthic macrofauna (Giani et al. 2012), by scraping the seafloor, disrupting bed-forms, re-suspending sediments and changing their size and texture (Collie et al. 1997; Pranovi 2000). These effects have been exacerbated by the modernisation of fishing technologies (GPS systems), enhanced capacity and catchability (Damalas et al. 2015); although there is evidence that they have been hindered by the lack of generational replacement in the trawling sector labour force (Buonfiglio et al. 2011).

Our knowledge of overexploitation of NAS’ fisheries is based on fairly recent data (Cardinale et al. 2017) and there is a lack of information on the long-term trends of species’ abundance and diversity. Trends in abundance of demersal stocks in the area, by the MEDITS International Trawl Survey, have only been monitored regularly since 1996, with the exception of two trawl surveys, one conducted in 1948–1949 and the other in the 1980s (Jukic-Peladic et al. 2001). Temporal changes in diversity of non-commercial benthic species have never been monitored by Italian fisheries agencies (Bastari et al. 2017) and trawlers’ effects on benthos’ diversity and seafloor integrity has only been investigated in the last three decades (Pranovi et al. 2000; De Biasi and Pacciardi 2008; Petovic et al*.* 2016). To this day, most of the benthic defaunation remains unrecorded and no historical baselines are available to compare the unexploited status of the seafloor (i.e. before the introduction of the *rapido*) to the present one. The lack of clear historical baselines of benthic diversity and seafloor integrity might have caused, among other factors, the failure of NAS’ *Good Environmental Status* (GES), an objective set by the European Commission’s (EC) Marine Strategy Framework Directive (MSFD) (Rice et al. 2012; Pita et al. 2020), aiming to mitigate the anthropogenic pressure exerted on the seafloor by 2020 (Rice et al. 2010).

The weaknesses in long-term scientific data collection of both target and non-target species (Carpi et al. 2017), impede the definition of baselines, leading to a misinterpretation of present conditions and a flawed formulation of effective conservation targets (Huntington 2000; Papworth et al. 2009). This issue is identified as Shifting Baseline Syndrome (SBS), corresponding to the erroneous perception of a current biological system due to the invalid information on its past conditions (Kahn and Friedman 1995). The involvement of fishing communities through Local Ecological Knowledge (LEK) represents a valuable instrument to integrate new information into the gaps left by conventional science.

LEK is defined as the collective environmental knowledge of local resource users (Beaudreau and Levin 2014), developed through human interaction with the natural environment and its biological components (Braga et al. 2018). When culturally transmitted through generations, the cumulative knowledge of LEK is more properly defined as Traditional Ecological Knowledge (TEK). Given the similarity between terms, TEK is often used as a synonym for LEK (Berkes 2012). When referring to fishing communities and fishers, the body of LEK is also regarded as Fishers’ Ecological Knowledge (FEK) (Neis et al. 1999a; Pita et al. 2020). The memory-based FEK has been adopted to recall past environmental conditions by devising indicators of the habitat’s pristine status, defined as historical baselines (McQuatters-Gollop et al. 2019). FEK has played a complementary role to traditional science systems by detecting a variety of long-term changes in: ecosystems’ structure (Viegas et al. 2016); fish catches (Beaudreau and Levin 2014; Coll et al. 2014; Martins et al. 2018) and benthic diversity (Bastari et al. 2017); and by providing information on species’ ecology and behaviour (Gaspare et al. 2015). The qualitative approach of FEK has improved fisheries management (Novacek and Cleland 2001; Barclay et al. 2017; McQuatters-Gollop et al. 2019) and has enabled scientists to enhance sampling and formulate new hypotheses for research (Silvano and Valbo-Jørgensen 2008). The use of FEK in marine policies has consequently enriched fishers’ social and political role, by involving them in decision-making processes and establishing a dialogue with stakeholders (Turner et al. 2000).

Given the need to provide baselines for marine management (Hind 2015) and the positive effects of FEK’s adoption in science and policy, the participation of fishing communities in decision-making processes has been promoted across multiple international bodies. In particular, the General Fishing Commission for the Mediterranean and Black Sea (GFCM) of the Food and Agriculture Organization of the United Nations (FAO) devised a Regional Plan of Action for Small Scale Fisheries in the Mediterranean and Black Sea RPOA-SSF (2018). This denounced the lack of recognition and involvement of resource users and proposed their enhanced participation in policy-making at a both national and international scale, by 2028 (GFCM 2018). With the publication of *Voluntary Guidelines for Securing Sustainable Small-Scale Fisheries* (VGSSFs) the FAO has also expressed the need to recognise fishers as “holders, providers and receivers of knowledge” and to support fishing communities’ knowledge as a valuable instrument to inform local governance (FAO 2015). Furthermore, the failure of the ECs Common Fisheries Policy (CFP) to achieve catches at sustainable levels by 2020 (Soma et al. 2018) has been associated with, among other elements, inadequate participation of resource users in devising management measures (Carpi et al. 2017).

Both the EC and FAO suggest enhancing FEK’s involvement in national and international management decisions. However, Italian fishers still lack a substantial representation in marine management policies. Local fishery cooperatives are still not incorporated in the country’s policy-making, delaying a social empowerment process already consolidated in other European countries (Buonfiglio et al. 2011). By building on previous studies of FEK in the Mediterranean (Coll et al. 2014; Damalas et al. 2015) and the Italian NAS (Azzurro et al. 2011; Bastari et al. 2017), the current study aimed to enhance the involvement of fishers’ knowledge in scientific research and policy making, in line with international guidelines for sustainable fisheries.

Despite being in constant contact with the marine ecosystem, fishers can also be prone to SBS (Turvey et al. 2010). The long-term depletion of the NAS’ ecosystem, coupled with the limited knowledge transmission between fishing communities’ members, might impoverish historical knowledge, especially of younger fishers. In fact, when younger observers are less aware of past environmental changes, a highly disrupted present ecosystem can instead be normalized as ‘healthy’ (Ainsworth et al. 2008). Since the start of the 21st Century, the Italian NAS fishery has witnessed a reduction in generational replacement (Buonfiglio et al. 2011) and a preference for less physically demanding and more economically rewarding vocations than trawling (Manfredi and Piccinetti 2011). This might have limited knowledge transmission relating to changes in local commercial demersal stocks, non-commercial benthic species and the benthic ecosystem. Differences in generational perceptions of the environment constitute symptoms of a rapidly changing environment and can be used to estimate long-term changes in the status of species and habitat. Nonetheless, future reliance on the current crop of young fishers to set historical baselines for the NAS could lead to incorrect conservation actions, representing a threat to the future of the Adriatic marine biota (Bender et al. 2014). The study aimed to contribute to knowledge of SBS in the NAS (Fortibuoni et al. 2016), by investigating the presence of SBS regarding target and non-target species’ diversity, benthic species’ diversity and sources of seafloor degradation.

The study focussed on three communities of trawling fishers along the Emilia Romagna region of the Italian NAS, and had two objectives:

i. To produce FEK-informed long-term regional trends of the four most fished demersal species: sole, common cuttlefish (*Sepia officinalis*), red mullet and mantis shrimp (Mulazzani et al. 2015). A quantitative approach was used, in order to better compare FEK-informed trends with regional catch reports. Given FEK’s ability to enrich traditional scientific knowledge, this objective aligned with EC and FAO’s request for more socially inclusive marine management policies.
ii. To investigate the presence of generational SBS regarding declines in: (a) target and non-target species diversity; (b) benthic diversity; (c) causes of, and solutions to, seafloor degradation. To achieve this, a qualitative approach was used as a well-established instrument for documenting change in communities’ environmental knowledge (Newing 2010).

## Materials and Methods

### Study area and fishery characteristics

**Fig. 1:**
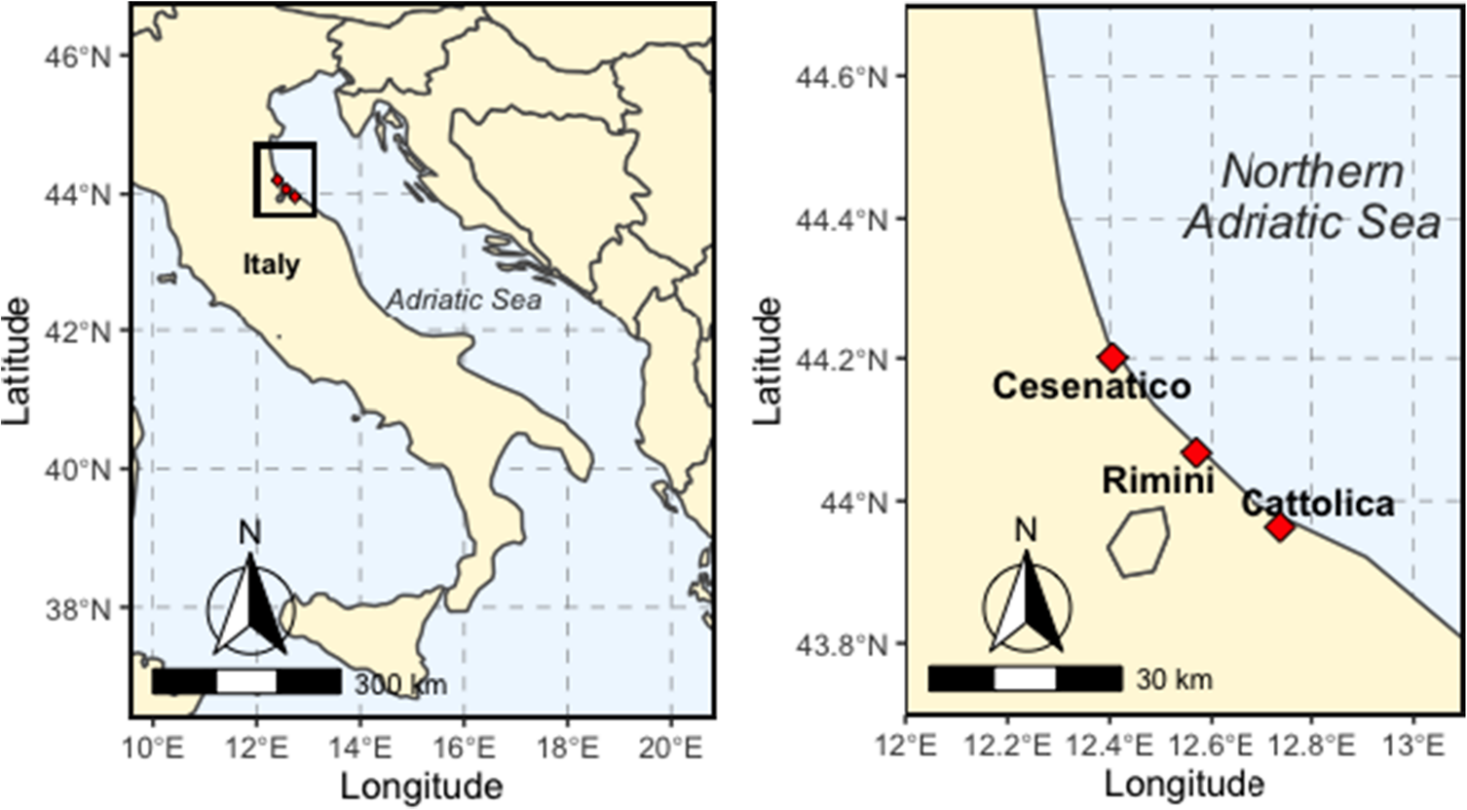
Map of Italy (left) showing the Adriatic Sea and the study area (black box). Expanded view of the study area showing the location of the three Italian ports Rimini, Cesenatico and Cattolica

The study was conducted in north-east Italy, along the southern coast of the Emilia Romagna region, in the ports of Cattolica, Rimini and Cesenatico. About fifty kilometres of shoreline separate the northern site, Cesenatico, from that in the south, Cattolica, with Rimini located between them (Fig. 1). In this area, the Adriatic Sea is characterised by maximum depths of 50 m (Rinaldi 2012) and by muddy-sandy sediments. The seabed in the first couple of kilometres from shore is made up of sand, followed by 3–4 km of silt and clay and subsequently by 40–45 km of mud (Rinaldi 2012). The benthic biota inhabiting the western side is composed of endofaunal species such as molluscs, polychaetes, epifaunal sponges, ascidians and anemones (McKinney 2007). Given the small inclination of the seafloor, the zonation of benthos’ distribution runs parallel to the coast (Manfredi and Piccinetti 2011).

All ports were characterised by the presence of local fishing cooperatives, with a total of 200 fishers in Rimini, 210 in Cesenatico and 279 in Cattolica. The port of Rimini accommodates 58 vessels, of which 29 are bottom trawlers. The port of Cesenatico has a total of 80 vessels and 25 of them are trawlers. Cattolica has the smallest trawling fleet of all, with a total of 81 vessels but just 2 working trawlers. The main bottom-trawling nets used in the three communities are the *rapido* trawl, the centuries-old *tartana* otter trawl, and the *americana* otter trawl.

### Data collection

A total of 53 fishers belonging to the trawling sector were interviewed in Rimini (*n* = 28), Cesenatico (*n* = 21) and Cattolica (*n* = 4), from June through August 2019. The total number of interviews was affected by temporal restrictions such as the month-long closed season at the end of summer. Given the male gendered predominance of fishing, no woman was found within the fishing communities, impeding any female representation in our study, with the exception of the interviewer. All fishers belonged to the local cooperatives: “Cooperativa Lavoratori del Mare” in Rimini, and “Casa del Pescatore” in both Cesenatico and Cattolica. Before the official interviews, a period of two weeks was spent amongst the fishing communities, talking to potential participants and conducting informal pilot interviews. This provided information of the local context and issues, the mastering of the local vocabulary regarding fish, benthic species, fishing grounds and fishing gears (Neis et al. 1999b) and a reciprocal building of trust (Newing 2010). Social entry and acceptance in the communities were facilitated by cultural brokers, that is, ex-representatives and/or renowned individuals of the fishing cooperative. The small number of interviewees conducted in Cattolica was due to the absence of any cultural broker for such port, while low numbers of novice interviewees represented a symptom of generational abandonment of fisheries (Buonfiglio 2011). In each town, the majority of fishers were approached along the harbours and in bars nearby the fishing cooperatives. A small number of elderly fishers were also approached in net repair buildings. Free, prior oral consent was requested before conducting structured interviews. The interviews consisted of both open and closed questions, with quantitative and qualitative answers, as recommended in Martins et al. (2018). All questions were asked in the same order, while easy and unthreatening questions were posed at the start, to spark fishers’ curiosity and minimize their mistrust (Newing 2010). When possible, the questionnaire was individually applied (Bender et al. 2014).

The questionnaire was composed of four sections. The first was designed to gather sociological characteristics of the interviewee, such as their age, the year they started fishing, the year they ended, their vessel horse power (hp), boat size (m) and type of net used. The second section consisted of a quantitative set of questions to trace the long-term trend in catch rates of sole, common cuttlefish, mantis shrimp and red mullet, the four most commonly landed species by Northern Adriatic bottom-trawlers (Mulazzani et al. 2015)*.* The decision of selecting the above species was linked to their historical presence within the fisheries, making them well recognised by all fishers (Turvey et al. 2010). Respondents were asked about the greatest catch, that is, the largest amount caught in a day (kg), and the year of that catch for each of the four species (as per Bender et al. 2014; Maia et al. 2018). To simplify the recollection of largest catch, the use of “*casse*” (i.e. boxes used to store and transport the fish) as a unit of measurement was adopted. Each *cassa* weighs between 4 and 7 kg, depending on the species. When the exact year of largest catch could not be recalled, the decade was then asked and its mean year was used for convenience (i.e. 1985 for the 1980s).

The third section consisted of free-lists regarding both target and non-target species, benthic species, and their changes over time (Giglio et al. 2014). This method was chosen to account for the possibility that fishers were not able to quantitatively measure changes in all commercial species (including species not caught by the interviewees), non-commercial species, and benthic by-catch. The interviewees were asked to list all the species they thought had decreased in abundance over time. Common names of benthic vertebrates and invertebrates varied among neighbouring communities, thus a taxonomic manual for local biodiversity was used to group the same species with different denominations and to facilitate the recognition of their scientific names. When specific species could not be recalled, family names were used for convenience.

The fourth and final section of the questionnaire involved a set of questions on environmental perception and ecological knowledge across generations (Bender et al. 2014). In particular, the questions aimed to investigate whether different generations had a similar consideration of the causes of, and solutions to, seafloor disruption, and whether the younger fishers had a particularly skewed perception of the benthic habitat. The respondents were asked about: (a) whether they perceived a disruption in benthic diversity compared to previous generations; and, if so, (b) what they thought was the main factor leading to seafloor degradation; and (c) what they believed to be the main solution to maintain seafloor integrity. The “Snowball” method (Martins et al. 2018) and chain referral (Newing 2010) were used to reach potential interviewees. An English translation of the questionnaire is provided in Appendix S1.

### Data analysis

Years of experience of the interviewees were categorised into three groups: novices (1–20 years, *n* = 13), experienced (21–40 years, *n* = 24) and veteran (> 40 years, *n* = 16) to ensure similar sample sizes (Maia et al. 2018). All statistical analyses were conducted with the open-access R software, using a significance level of probability *P* = 0.05.

For the trend in catch rates of the four commercial demersal species, greatest catch (in kg) was plotted as a function of effort. The effort was expressed as vessel power- transformed from hp to kw units, multiplied by the average daily fishing duration (h). The use of vessel power as the only variable affecting fishing efficiency over time comes from the decision to favour simplification over potential memory bias. Questions on changes of gear size and efficiency were initially added to the questionnaire, but soon excluded due to the high uncertainty of the answers collected. Aware of vessel power’s ability to encapsulate the modernisation of the fishing industry we were also conscious of the consequences of such simplification on the validity of catch rates trends. The resulting catch-per-unit-effort (CPUE) [kg (kw h)^−1^] was analysed over time (year) for each interviewee (Bender et al. 2014). Given the potential non-linear dependence of catch rate over years, a generalised additive model (GAM, package ‘mgcv’ in R software) with a smooth function was applied to investigate significant changes in species catch rates over time (Martins et al. 2018). The four datasets (mantis shrimp, sole, cuttlefish and red mullet’s greatest catches) were tested for normality before engaging in statistical analyses. One substantial outlier recurring in every dataset was excluded, given the uncertainty in measurements expressed during the interview (i.e. the interviewee referred to the amount of *casse* caught for each fishing event rather than a full day of fishing).

To investigate the presence of SBS, two datasets were derived from the free-lists: one for commercial species and one for benthic invertebrates’ decline. The number of overexploited species listed by each fisher was plotted against the fisher’s years of experience and the three experience groups (i.e. novice, experienced and veterans) were graphically discerned. The two datasets satisfied the assumptions of a general linear model (GLM), run using the function ‘aov’ in R. The mean number of species mentioned was then yielded for each experience group. Normality tests were conducted before statistical analyses, which tested the null hypothesis that there was no difference in the number of species listed as depleted among the three groups of experience. Finally, to assess shifts in environmental perception among generations of fishers, as well as general biological knowledge of the interviewees, a set of tables were created (Giglio et al. 2014). Each table showed percentages of answers given by novice, experienced and veteran fishers regarding (a) the main cause of seafloor degradation; (b) a potential solution to seafloor degradation. Single answers to each of the above questions were gathered for every interviewee. For each experience group, identical answers were summed and transformed into percentages, to facilitate comparison with the other groups.

### Limitations

General limitations of this approach should be considered. Firstly, when seldom interviewed in socially relevant scenarios (e.g. the fishers’ bar), the presence of an audience surrounding the interviewee represented a potential bias, due to the inclination of spectators to ‘help’ the respondent answering the questions. Secondly, initial mistrust was occasionally sensed. This often stemmed from the perceived image of the interviewer as a scientist, often regarded as having opposite interests to those of fishers. In contrast to this, an opposite behaviour was often embraced, given the female gender of the interviewer. Interest increased facilitating interviews, whereas sexual jokes and insinuations occasionally arose. The use of a structured questionnaire and the lack of recordings might have limited the collection of important informal insights (Johannes et al. 2000).

## Results

The 53 interviewees ranged from 18 to 84 years of age and from 2 to 51 years of experience, with the oldest year of starting activity dating back to 1952. Forty interviewees (75%) revealed fishing to be a family activity. When assessing interviewees’ fishing history, a temporal trend toward longer vessels (from a minimum of 8 m to a maximum of 30 m) and increased vessel power (from 24 to 1500 hp) was evident. *Tartana, americana* and *rapido* were the main trawling gears used by the respondents, few of whom also worked in the pelagic sector.

### Trends of demersal fish abundance

**Fig. 2:**
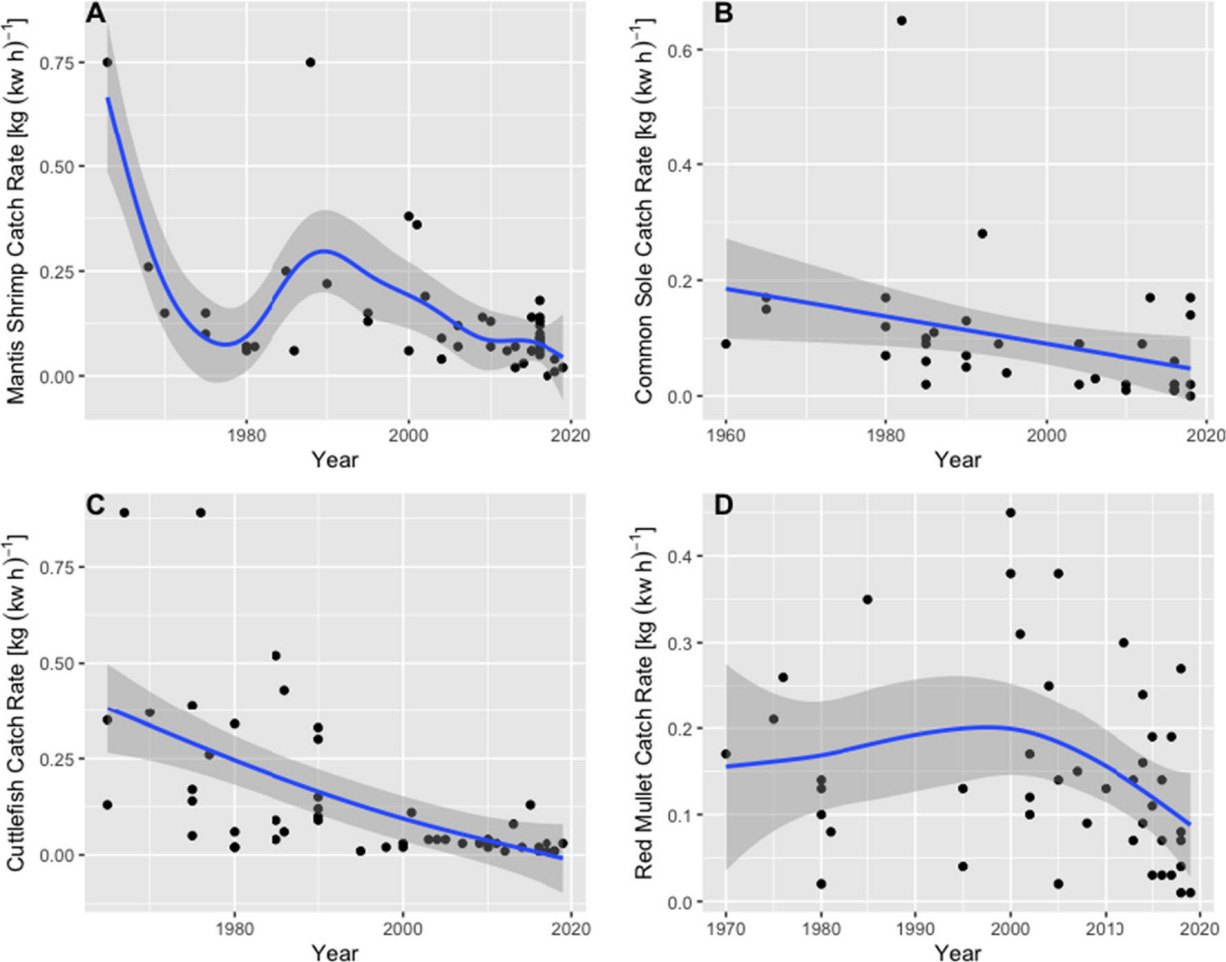
Catch rates [kg (kw h)^−1^] of four commercial demersal species by year, obtained from fishers’ memory of species’ best catch. The Generalised Additive Model (GAM) fits for mantis shrimp (*Squilla mantis*) (**a**), sole (*Solea solea*) (**b**) and cuttlefish (*Sepia officinalis*) (**c**) (in blue, with 95% confidence interval, shaded grey) were all significant (respectively: df = 45, Gaussian smoother, *P* = 3.53e−06; df = 35, Gaussian smoother, *P* = 0.03; df = 46, Gaussian smoother, *P* = 2.98e−05). The GAM fit for red mullet (*Mullus barbatus*) (**d**) was not significant (df = 44, Gaussian smoother, *P* = 0.15)

The long-term trends of species’ catch rate exhibited different patterns with different species. According to FEK, common cuttlefish catch rates decreased almost linearly over the 54 years (Fig. 2c), from an average rate of 0.24 [kg (kw h)^−1^] in 1965 to one of 0.03 [kg (kw h)^−1^] in 2019. A significant decline was also estimated for sole (Fig. 2b). Catches of mantis shrimp declined, then rose slightly in the 1980s, but have since dropped, accounting for a significant decline (Fig. 2a). The null hypothesis for no change in the catch rates of cuttlefish, sole and mantis shrimp could thus be rejected. By contrast, no significant change in the catch rates of red mullet was detected (Fig. 2d).

### Generational accounts of species’ diversity

**Fig. 3:**
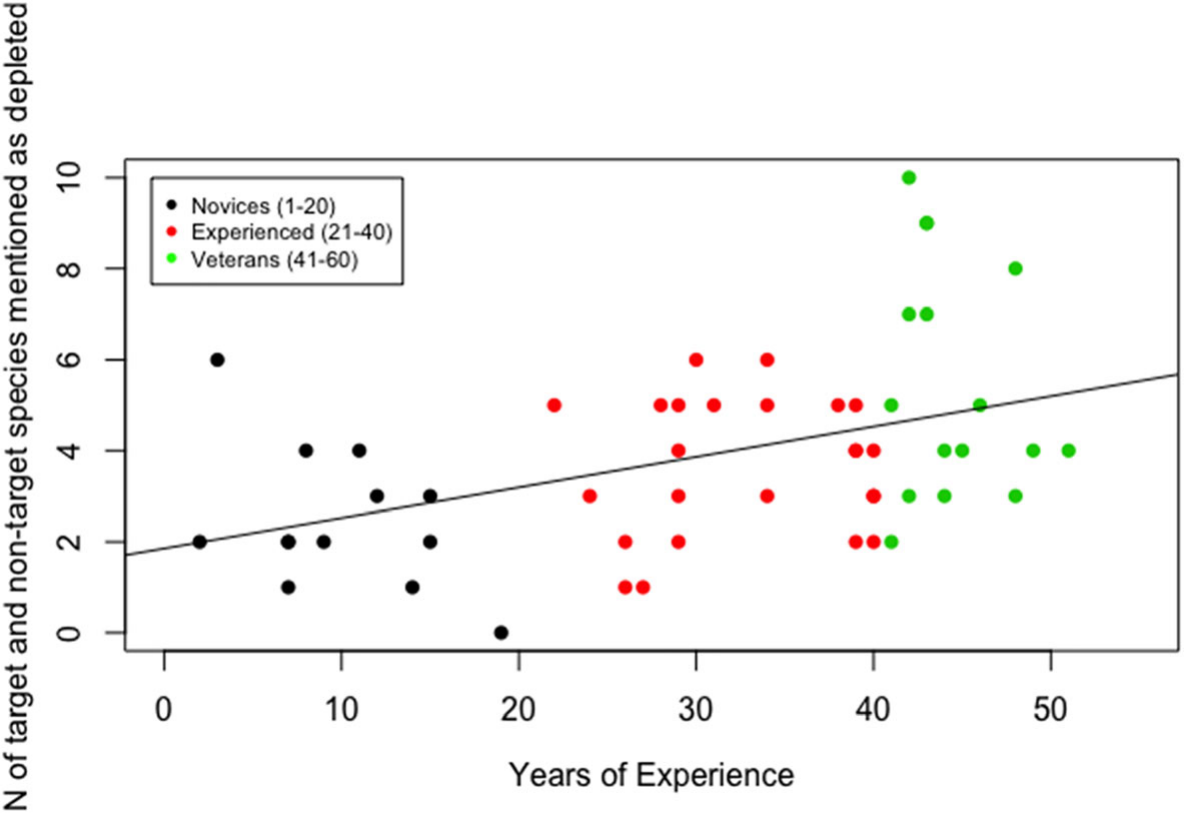
Number of target and non-target species mentioned as depleted by fishers against years of their experience. The fishers were grouped in three categories (i.e. novices in black with 1 to 20 years of experience; experienced in red, with 21 to 40 years of experience; and veterans in green, with 41 to 60 years of experience)

**Fig. 4:**
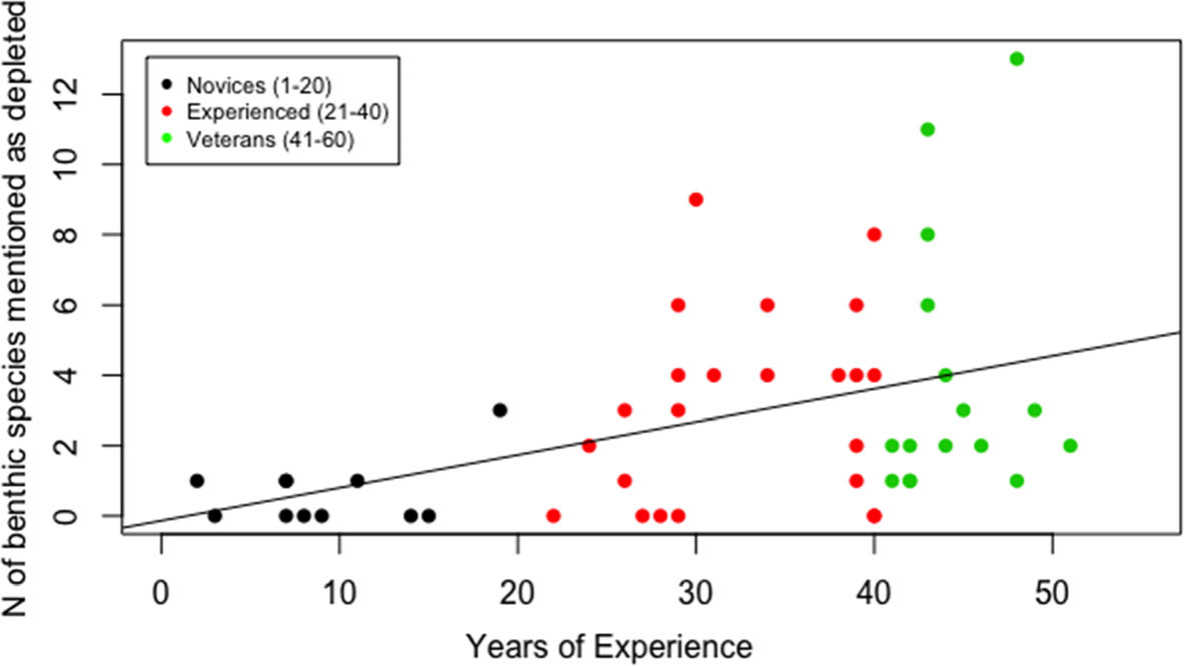
Number of benthic species mentioned as depleted by fishers plotted against years of their experience. The fishers were grouped in three categories (i.e. novices in black with 1 to 20 years of experience, experienced in red, with 21 to 40 years of experience and veterans in green, with 41 to 60 years of experience)

The number of depleted commercial species listed revealed a positive correlation with fishers’ years of experience (Fig. 3; ANOVA, Adjusted *R*^2^ = 0.17, *P* < 0.002). An average of 2.5 species were identified as depleted by novices, 3.7 by experienced and 5.4 by veteran fishers. The most recalled species were: the common cuttlefish (*n* = 26), the common squid *Loligo vulgaris* (*n* = 25) and the Norway lobster *Nephrops norvegicus* (*n* = 18). A total number of 2 fish species were uniquely remembered by novices, whereas 8 and 12 species were solely remembered respectively by experienced and veteran fishers. The numbers of benthic invertebrates cited as declined also yielded a significant increase with gained experience (Fig. 4), rejecting the null hypothesis for no difference in number of species mentioned among groups (ANOVA, Adjusted *R*^2^ = 0.17, *P* < 0.002). Novices mentioned an average of 0.6 invertebrates’ species as depleted, experienced cited 3.1 and veteran 3.9. 46% of novices indicated that no benthic species had declined compared to 40% of experienced fishers. On the contrary, all veteran fishers listed at least one species as depleted. The most mentioned species was the *Holothuria* spp. (*n* = 21), followed by the starfish *Asteroidea* spp. (*n* = 19), the fan mussel *Pinna nobilis* (*n* = 16) and the sponge *Geodia cydonium* (*n* = 16). No species were mentioned solely by novices, while the experienced and veteran groups remembered respectively 11 and 15 species that no other group recalled.

### Generational accounts of seafloor’s degradation

85% of all interviewees expressed their opinions on the causes of seafloor degradation in the Northern Adriatic (Table 1). High fishing effort was the first cause of seabed disruption according to 70% of novices and 50% of experienced, followed by pollution (recalled by 10% of novices and 20% of experienced). The majority of the veteran fishers considered the use of *rapido* to be the primary cause of seafloor degradation (47%), followed by high fishing effort (33%). Neither pollution, climate change nor plastic was cited by older fishers, but these factors were recalled by at least one participant from the novice group. On the contrary, no young observer stated *rapido* to be the primary cause of benthos disruption.

**Table 1:** Causes of seafloor degradation according to fishers. Percentages of answers were produced for every category of experience (i.e. novices, experienced and veterans)

Causes of seafloor degradation	Novices	Experienced	Veterans
High fishing effort	70	50	33
Use of *rapido*	0	15	47
Pollution	10	20	0
*Rapido* and fishing effort	0	5	7
Fishing effort and pollution	0	0	7
Marine currents	0	5	6
Climate change	10	5	0
Plastic	10	0	0

82% of informants answered the question posed on possible solutions to seafloor degradation (Table 2). A considerable percentage of respondents had no solution to tackle seabed disruption, but this was more prominent in the novice group (30%). Around 26% of experienced fishers declared the protection of the seabed to be achieved primarily through limiting vessel power, whereas the majority of veteran observers (33.3%) indicated that the use of *rapido* gear should be limited or stopped, followed by a limit to fishing effort (26.7%). None of the novice fishers stated that *rapido* should be stopped for the maintenance of seafloor’s integrity.

**Table 2:** Solutions to seafloor degradation according to fishers. Percentages of answers were produced for every category of experience (i.e. novices, experienced and veterans)

Solutions to seafloor degradation	Novices	Experienced	Veterans
Limit fishing effort	10	0	26.7
Stop or limit use of *rapido*	0	15.8	33.3
Creating closed areas	10	0	20
Limit vessel power	0	26.3	13.3
Limit river run-off	10	16	0
Limit days at sea	10	16	0
Vessel demolition	10	5.3	0
Landing by-catch	10	0	0
Longer closed season	10	5.3	0
Change gears	0	5.3	0
None	30	10.5	6.7

## Discussion

### Trends of demersal fish abundance

The long-term trends inferred in this study, measuring the CPUE of cuttlefish, sole, mantis shrimp and red mullet, might provide a more accurate view of species’ abundance than the currently available official fisheries’ data (Huntington 2000). Indeed, while this study adopted CPUE—in [kg (kw h)^−1^]—to assess trends in commercial demersal species’ abundance, national datasets commonly use the measure of landings only (in kg). Landings do not account for any measurement of effort (i.e. vessel power, days at sea, gear type, hours of activity) (Pauly et al. 2013), which has changed substantially in recent years (Damalas et al. 2015). Given the discrepancy in units of measurements, a comparison between FEK and national datasets is nearly impossible to make. Indeed, only FEK’s declining trend of common cuttlefish is analogue to the species’ national dataset of annual landings (Lotze et al. 2011; UNIPD 2018), while sole and mantis shrimp present increasing trends in official landings (UNIPD 2018), opposite to FEK’s datasets. Despite being a more accurate measure of abundance than landings, CPUE might still not ensure a proportional measurement of true abundance (Harley et al. 2001). In particular, the validity of CPUE might be impaired by both the subjectivity of fishers’ knowledge (Beaudreau and Levin 2014) and the study’s methodology. Fishers’ inability to remember the length and width of the net (m) and its mesh size (mm) may have impeded the estimation of a more accurate CPUE, while the decision to simplify fishing effort by only using vessel power (thus excluding several variables such as GPS introduction, changes in gear type and size etc.) represents a potential source of error for catch rates trends. The unequal distribution of points across time and the higher variability of points in the past (Fig. 2a–c) might be caused by the smaller number of interviewees with past experience and by a disrupted memory of events happening further in the past, respectively (Azzurro et al. 2011). Furthermore, interviewees’ recalling of a particularly rich fishing day might have resulted in the erroneous assumption of a peak year, as may have been the case of mantis shrimp (Fig. 2a). The use of *casse* as a unit of catch quantity assisted the recollection of the greatest catch, but may not be very precise.

In addition to the quantitative descriptions of catch rates, fishers added information about the behaviour of the demersal species sole and mantis shrimp*,* to provide some context for when the greatest catches took place. As multiple fishers stated, the most profitable days for landing sole were related to hypoxic events, during which fish tended to aggregate in oxygen-rich areas, facilitating catches. Studies have shown a link between hypoxic events and the physiology (Viall 1997), stress levels and distribution of demersal fish (Pihl et al. 1991), with species migrating from deeper to shallower waters. Fishers also linked the richest catches of mantis shrimp to storms events, describing the tendency of shrimps to ‘escape’ their burrows during rough seas. The emergence of bottom-dwellers has been shown to be connected to storms’ disruption of burrows in shallow waters (Mulazzani et al. 2015). In particular, the best catch recalled by numerous fishers in the year 2016 (Fig. 2a) was linked to a storm phenomenon resulting in large landings of mantis shrimp.

The lack of a clear trend in the red mullet’s CPUE (Fig. 2d) might be indicative of the absence of long-term change of abundance of the species.

### Generational accounts of species’ diversity

The significant discrepancy of perceptions between old and young fishers provides evidence of SBS in relation to both target and non-target species and benthic species’ diversity. The SBS of novice fishers might be due to the long-term, fast-paced marine defaunation (Barausse et al. 2011; Fernández-Llamazares et al. 2015), preventing younger generations from encountering an equal number of species as their older counterparts. The short experience of novices might induce them to perceive short-term increases in abundance of particular species as sustainable, without taking into consideration the overall long-term decline.

When conducting the free-list for target and non-target species, the veteran group was the only one to cite elasmobranchs (i.e. starry skate *Raja asterias,* small-spotted catshark *Scyliorhinus caninula* and common smoothhound *Mustelus mustelus*) as declining over time. This is in line with recent studies which highlight an 80% decline in elasmobranchs landings from 1945 to 2012 (Barausse et al. 2014), with *R. asterias* and *M. mustelus* respectively listed as nearly threatened, (NT) and vulnerable (VU) in this region by the International Union for Conservation of Nature (IUCN) Red List (Farrell and Dulvy 2016; Serena et al. 2016; Fernandes et al. 2017). Various species listed as declined by veteran (i.e. *Scorpaena porcus, Arnoglossus laterna, Illex illecelebrosus*) and experienced fishers (i.e. *Zeus faber, Chelidonichthys lucerna, Umbrina cirrosa* and *Trigloporus lastoviza*) contradict the IUCN criteria, which declares them of Least Concern (LC) of extinction. This might be due to the discrepancy of geographical ranges between the IUCN assessment, assessing species at the European scale (Nieto et al. 2015), and FEK, focussing on a specific region. Given FEK’s efficiency in reporting regional depletions (Coll et al. 2014) its implementation in local conservation policies should be taken into account. Finally, the pelagic species of European pilchard (*Sardina pilchardus*), *round sardinella* (*Sardinella aurita*) and European anchovy (*Engraulis encrasicolus*) were listed as overexploited by both veteran and experienced fishers, as supported by scientific assessments (Cardinale et al. 2017). The failure of novices to recall all the above species might represent a further demonstration of their decline.

The majority of fishers interviewed referred to the benthic by-catch species as “*sporco”* (literally translated as “dirt”). Such a designation gives insights into respondents’ perception of benthic fauna, perceived to exert a negative impact on catches by rendering fish commercially unsellable. It also explains the lower average number of benthic species listed (2.6) compared to commercial species (3.9), given that species of no commercial value are less likely to be remembered. Fishers found it easier to recall large benthic species or species exerting particular features. When naming benthic fauna, an association with terrestrial plants was often made by experienced and veteran fishers (e.g. ‘orange’, ‘date’, ‘cucumber’, ‘tangerine’, ‘tomato’) and the seabed described as a ‘cultivated field’. The latter contrasted with the account of a novice fisher, depicting the Adriatic seafloor as a ‘naturally marine desert’. Such discrepancies in the generational perception of the environment are also visible in fishers’ accounts of benthic diversity, with novices recalling an average of fewer than one species and veteran fishers recalling an average of four. This might be linked to a recent anthropogenic defaunation of the benthic habitat, coupled with a lack of information exchange. Overall, the perceived decline of three of the most listed species *Holothuria* spp., *Geodia cydonium* (not evaluated within the IUCN Red List) and *Pinna nobilis* (listed as Critically Endangered, CR) (Kersting et al. 2019) is similar to accounts of fishers in the neighbouring region of Marche (Bastari et al. 2017). The reduction in *P. nobilis*, regarded as a threatened species by the Specially Protected Areas and Biological Diversity Protocol of the Barcelona Convention (BC) (UNEP 1999), remains of particular concern. Of the species recalled by fishers, the BC’s protocol (Annex II), has also considered the date mussel *Lithophaga lithophaga* and *G. cydonium* as endangered species and the European spider crab *Maja squinado* as a species whose exploitation is under regulation (Annex III). Other species such as *Asteroidea* spp., *Othuroidea* spp., *Echinoidea* spp. and *Dromia personata* were also listed as depleted by fishers, but not found on international lists of threatened species. Further studies should therefore be conducted to verify the status of the above species.

Potential errors when conducting free-lists need to be accounted for. The variety of unofficial names used by fishers, often depicting the same species, represented an issue when conducting the taxonomical identification. Given the uncertainty arising among interviewees when matching a species listed with its official name, species pertaining to the same family were grouped together (as with *Olothuria* spp. and *Asteroidea* spp.). This might have impaired the identification of specific individual species as depleted.

### Generational perceptions of seafloor’s degradation

SBS appears to also have affected generational perceptions on causes of, and solutions to, seabed disruption. Differences in respondents’ perception might be linked to fishers’ participation (or lack of it) in the pivotal historical events of local fisheries. Veterans, who lived through the advent of *rapido* (1950s) recalled it as the main disruptor of benthic fauna, while experienced and novice fishers mostly linked the disruption of the seabed to increased fishing effort, given their lived experience of the modernisation of the local fleet (1980s). Furthermore, novices’ increased awareness of pollution, plastic and climate change as pressures on seafloor constitute another example of how an individual’s place in history can shape knowledge of current events. Although not excluding the pressure of numerous factors responsible for seafloor’s disruption, studies have shown intensive trawling to represent the major cause of benthic disruption (Barausse et al. 2011; Sciberras et al. 2018). Therefore, both the use of *rapido* and the increase in fishing effort are probable causes of changes in the benthic habitat.

The inability to provide answers to seafloor degradation increased with decreasing experience (30% of novices did not answer the question, compared to 10.5% of experienced and 6.7% of veterans) and may be related to a lack of interest (Bastari et al. 2017), a lack of knowledge, or to the belief that the seabed still constitutes a healthy ecosystem- as stated by some novices. The incapacity of novices and experienced fishers to provide unanimous and sound solutions for seafloor’s protection might constitute a sign of SBS. In particular, no novice suggested limiting the use of *rapido* as a solution to seafloor’s degradation. This might be linked to novices’ inability to question already established techniques as detrimental for the marine environment. On the contrary, veterans witnessing the advent of bottom-trawlers might more easily compare the ecosystem status prior to *rapido* to the one immediately after its adoption.

## Conclusion

This study showed the ability of fishers to provide sound knowledge on historical, regional trends of abundance and marine biodiversity, often exceeding national and international scientific datasets (Saenz-Arroyo et al. 2005). In common with other studies we recommend an enhanced participation of marine resource users in both science and policy sectors (Gelcich et al. 2008; Santiago et al. 2015). Sharing the responsibility of managing natural resources with local users enhances fishers’ livelihoods by valuing their political viability and reducing dissatisfaction towards the establishment (Gaspare et al. 2015). It also allows for a more complete understanding and management of the local marine resources (Braga et al. 2018). Scientists should therefore invest in relationship building with communities, involving them in research, from discussion of design to the sharing of results (Stevenson 1998; Brook and McLachlan 2008). To enhance an open and bidirectional dialogue with informants, the adoption of social research techniques by conservationists is necessary (Huntington 2000).

Given the presence of a SBS, hindering the efficacy of FEK in advising marine policies, the collection of past information through photographs, reports, films and recordings (McClenachan 2009; Bender et al. 2014), would facilitate the recalling of historical baselines by younger fishers. Particular importance should be given to knowledge experts, such as veteran fishers, by enhancing the transfer of anecdotes to younger generations through community meetings and informal discussions (Turvey et al. 2010).

FEK represents an insightful instrument for conservation, and resource users’ voices must be recognised and prioritised to ensure the long-term sustainability of fishing in overexploited areas such as the Mediterranean.

## Electronic supplementary material

Below is the link to the electronic supplementary material.

Electronic supplementary material 1 (PDF 132 kb)
