# Supplementary material for: Fishers’ knowledge detects ecological decay in the Mediterranean Sea
Source: Ambio. 2021 Jan 16;50(6):1159–71. doi: 10.1007/s13280-020-01452-3 (PMC8068750; doi:10.1007/s13280-020-01452-3)
Supplement: Supplementary file 1 — Electronic supplementary material 1 (PDF 132 kb) [file 13280_2020_1452_MOESM1_ESM.pdf]

Ambio

Electronic Supplementary Material

This supplementary material has not been peer reviewed.

Title: Fishers' Knowledge Detects Ecological Decay in the Mediterranean Sea

Authors: Benedetta Veneroni, Paul G. Fernandes

QUESTIONNAIRE

Number: __ Date: ______ Place:______ Sector: __________

Name:______________

Age:____

Year of starting activity:________

Year of ending activity/still in activity:________

1. Is fishing a family activity for you?________

2. **Main Gear**

	From 60's to 70's	From 70's to 80's	From 80's to 90's	From 90's to 2000's	From 2000's to 2010's	From 2010's to present
**Vessel size** (m)						
**Type** (local name)						
**Length and width of the net** (m) (mean size)						
**Vertical opening** (m)						
**Mesh size (+cod end)** (mm)						
**Vessel power (**hp**)**						

3. How would you describe the current conditions of fish stocks regarding abundance?
( ) unaltered ( ) declined ( ) augmented

4. Do you know any overexploited species by fisheries in this region? ( ) Yes ( ) No

5. If Yes, which species? _______________

6. What was the largest amount of sole that you captured in a day (in *casse*)? __________

7. When?_____________

8. What was the largest amount of cuttle fish that you captured in a day (in *casse*)? __________

9. When?______________

10. What was the largest amount of mantis shrimp that you captured in a day (in *casse*)? ______

11. When?_____________

12. What was the largest amount of red mullet that you captured in a day (in *casse*)? ________

13. When?______________

14. Please, list the names of all the benthic species you

    remember:____

15. Of all these species, which ones you think have decreased in

    numbers?____

16. Do you think that the past generations of fishermen were able to witness a seabed richer in

    benthic species?

    () yes () no () I don't know

17. If yes, what do you think is the main cause for a disruption in the benthic

    habitat?____________________________________

18. In your opinion, what would be a solution to such disruption?_________________________

19. In your opinion, how important is seafloor integrity for the maintenance of abundant demersal fish? Tick **one** of the following boxes.

    - ☐ Not important
    - ☐ Relatively important
    - ☐ Really important
    - ☐ Essential
    - ☐ I don't know/ I cannot answer
